# A huge chest wall angiomatosis with pleural and rib invasion: A case report

**DOI:** 10.1097/MD.0000000000032242

**Published:** 2022-12-09

**Authors:** Yu-Chi Wang, Yun-Nan Lin, Chee-Yin Chai, Hsien-Pin Li, Yi-Ting Chen, Yur-Ren Kuo

**Affiliations:** a Division of Plastic and Reconstructive Surgery, Department of Surgery, Kaohsiung Medical University Hospital, Kaohsiung, Taiwan; b Department of Pathology, Kaohsiung Medical University Hospital, Kaohsiung, Taiwan; c Division of Thoracic Surgery, Department of Surgery, Kaohsiung Medical University Hospital, Kaohsiung, Taiwan; d Faculty of Medicine, College of Medicine, Kaohsiung Medical University, Kaohsiung, Taiwan; e Department of Biological Sciences, National Sun Yat-Sen University, Kaohsiung, Taiwan; f SingHealth Duke-NUS Musculoskeletal Sciences Academic Clinical Programme, Singapore.

**Keywords:** angiomatosis, case report, chest wall invasion, chest wall tumor, soft tissue tumor, vascular proliferation

## Abstract

Angiomatosis is a rare non-neoplastic proliferative vascular lesion that typically develops during childhood or adolescence with a female predominance. Management of angiomatosis is challenging because of the manifestation of a wide variety of lesions as well as their invasive and highly recurrent nature.

**Patient concerns:**

We report the case of a 74-year-old man who presented with a right lower back mass that persisted for a decade. The mass progressively enlarged and had been painful in the previous month.

**Diagnosis:**

Computed tomography (CT) revealed suspected lipomatous sarcoma with invasion of the ribs, pleurae, and lung parenchyma. The final pathological examination revealed angiomatosis.

**Interventions:**

The patient underwent wide composite excision of the tumor along with excision of the pleura and lung nodules in the right lower and middle lobes via video-assisted thoracoscopic surgery (VAST). Fasciocutaneous rotational flap reconstruction was performed immediately after the wide composite excision and video-assisted thoracoscopic surgery (VAST).

**Outcomes:**

The patient recovered uneventfully, was discharged without complications, and tolerated the daily activities well.

**Lessons:**

Angiomatosis is a rare benign vascular tumor that frequently mimics malignancy. Even if the patient profile does not match the reported epidemiology of this disease, differential diagnosis should be considered. Complete resection is the mainstay of treatment for the prevention of recurrence.

## 1. Introduction

Angiomatosis is characterized by diffuse proliferation of vessels and adipose tissue and has been reported to occur in a variety of locations, including the extremities, muscles, bones, and, in a few cases, the chest wall.^[1–3]^ Angiomatosis is a slow-growing mass that can mimic vascular malformations, soft-tissue tumors, or malignancies. It is more common in females in the first 2 decades of life.^[1]^ This case report discusses a rare case of lower back angiomatosis with invasion of the underlying ribs and pleura in an elderly male patient.

## 2. Case presentation

A 74-year-old man presented to our hospital’s clinic in the reconstructive and plastic surgery department complaining of a large mass in the right lower back region for the past 20 years. The main reason for his visit was the recent progressive enlargement of the mass for 1 month. The mass in the right lower back was accompanied by ecchymosis and positional pain. The patient had no medical, surgical, or trauma history.

**Figure 1.**
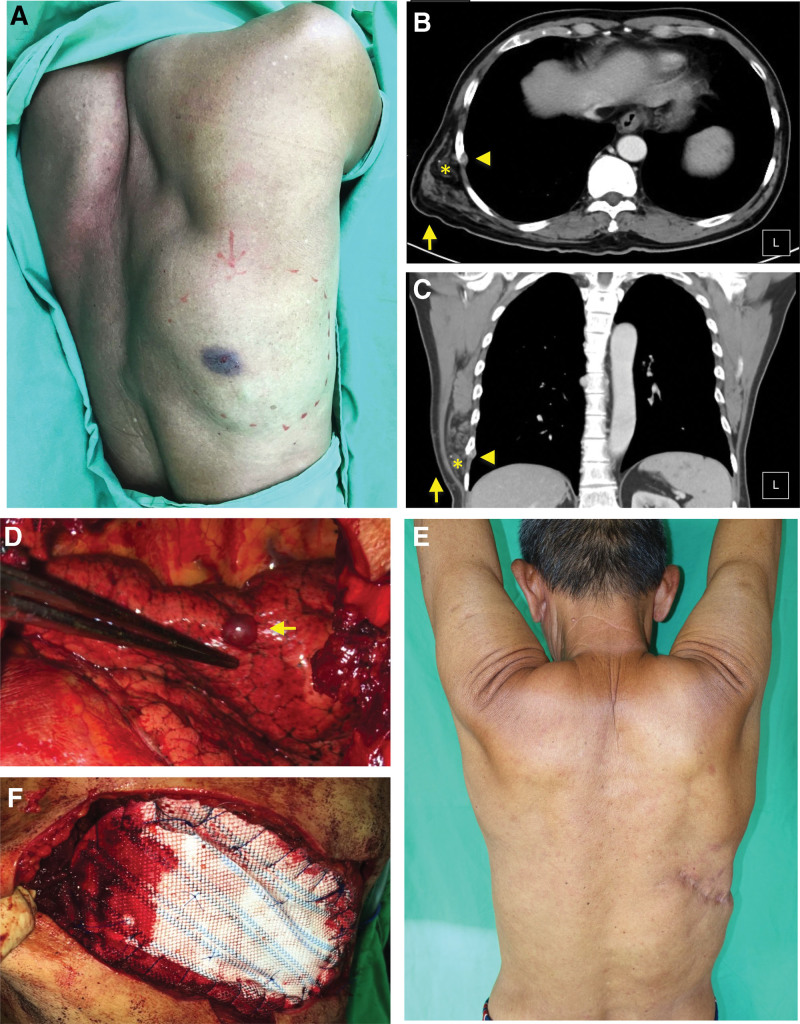
(A) A painful soft mass with a maximum diameter of 14 cm in the right lower back region with central bruising. (B and C) Contrast-enhanced CT scan images revealed a right chest wall mass (arrow) with calcification (yellow asterisk) and invasion to the right 7th, 8th ribs and pleurae (arrow head). (B) Axial view. (C) Coronal view. (D) Intraoperative image of hemorrhagic sponge-like vascular tissues were seen extensively involving the latissimus dorsi, ribs, pleurae and lung parenchyma (yellow arrow). (E) Intraoperative image of the polypropylene mesh for rib and pleura defects. (F) The follow-up results at 3 years postoperatively. No functional disability was observed. CT = computed tomography.

Physical examination revealed an ill-defined, soft, and tender mass with a maximum diameter of 14 cm and ecchymosis of the right lower back (Fig. 1A). No active bleeding and bruits on auscultation from the lesions. Laboratory examination results were within the normal range. A computed tomography (CT) scan revealed a 14.0 cm × 9.0 cm × 4.2 cm heterogeneously enhanced lesion with calcification, invasion of the 7th and 8th ribs, multiple concomitant nodular pleural-based and lung parenchymal lesions, raising the possibility of lipomatous sarcoma (Fig. 1B and C). No evidence of distant metastasis was observed.

**Figure 2.**
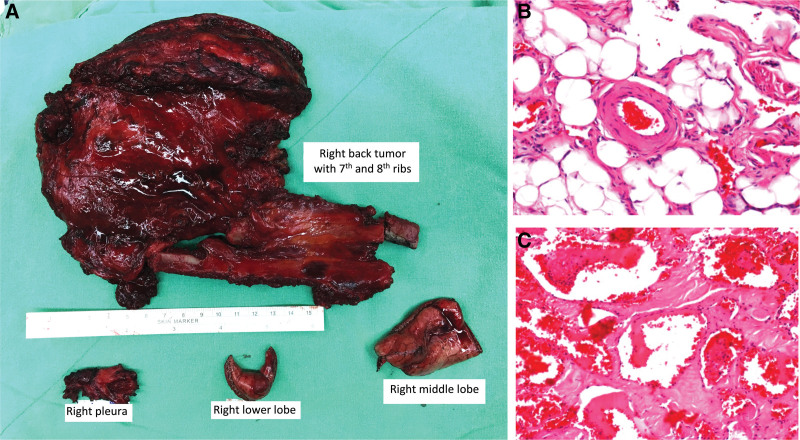
(A) The gross image of right lower back angiomatosis post wide excision, including right 7th and 8th ribs, right pleural tissue, as well as the right middle and lower lobes of the lung tissues. (B and C) Hematoxylin and eosin staining showed a typical angiomatosis lesion with disorganized vessels and small capillaries in (B) the mass lesion and (C) ribs. Magnification at 200×.

The patient underwent composite excision with 2 cm margins from the mass in the latissimus dorsi (16.0 cm × 11.0 cm), 7th (10.0 cm), and 8th (15.0 cm) ribs. Video-assisted thoracoscopic surgery (VATS) was performed to excise the right pleura and lung nodules in the right lower and middle lobes (Figs. 1D and 2A). The final defect size was 16.0 cm × 11.0 cm. The pleural and rib defects were repaired using polypropylene mesh (PROLENE®) (Fig. 1E). A fasciocutaneous rotational flap with an S-shaped design was used for the reconstruction of the skin and soft tissue defects.

Frozen sections from intraoperative excisional biopsy revealed no malignancy or clear surgical margins. Pathological examination revealed the smattering of large vessels and small capillaries throughout the soft tissue and bony structures (Fig. 2B and C). Immunohistochemical staining suggested no evidence of liposarcoma, with negative staining for cyclin-dependent kinase 4 (CDK4), mouse double minute 2 homolog (MDM2), and p16. These pathological findings were consistent with an unexpectedly rare diagnosis of angiomatosis.

The postoperative course was uneventful. Before discharge, the patient stayed in the surgical intensive care unit for 5 days and in the ward for 3 days, and an incentive spirometer was provided for pulmonary training. The patient tolerated the procedure without complications and recovered well with sustained daily activities (Fig. 1F). No recurrences were detected during the 3 years of follow-up.

## 3. Discussion

Angiomatosis is a benign vascular lesion that most commonly affects females during the first 2 decades of life.^[1]^ It can affect large segments of the body in a contiguous manner by involving several different tissue types and anatomical sites, although it usually involves the lower extremities.^[1–4]^ The etiology is congenital or acquired, with the former being phosphatase and tensin homolog (PTEN)/ phosphoinositide 3-kinases (PI3K) mutations or associated with Klippel–Trenaunay syndrome, Sneddon syndrome, or Gorham disease. The latter is acquired from infections, such as human immunodeficiency virus or bartonellosis.^[5,6]^ Radiological imaging may reveal nonspecific heterogeneous lesions in different tissue types with vascular involvement.^[6]^ Histopathological features include the proliferation of blood vessels of varying sizes, which are usually associated with large amounts of adipose tissue.^[1]^

This was an unusual case of angiomatosis in an elderly man with no relevant medical history. In addition, computed tomography (CT) revealed a heterogeneously enhanced lesion with extensive fat and focal calcification, primarily involving the lower back, with skeletal and pleural invasion of the chest wall, which are infrequent sites of angiomatosis. As a result, a clinical picture of malignant soft tissue tumor, such as liposarcoma, was highly suspected.

The definitive treatment of angiomatosis requires complete resection due to the high recurrence rate of up to 88%.^[1,3]^ 36% of patients demonstrate more than 1 recurrence during follow-up.^[1,5]^ Wide surgical excision is the mainstay of treatment for disseminated angiomatosis. Radiotherapy, embolization, interferon α2a adjuncts, and angiogenesis inhibitors have been reported in the literatures.^[1–3]^ In this patient, no local recurrence at 3-year follow up was observed, emphasizing the importance of margin-free complete resection.

In conclusion, angiomatosis is a rare soft tissue tumor that appears clinically similar to a variety of lesions such as hemangiomas, angiolipomas, liposarcomas, and metastatic tumors. Clinicians should consider angiomatosis as a differential diagnosis even if the patient profile does not fit the reported epidemiology of this disease. Although complete resection is challenging, particularly for extensively disseminated lesions, margin-free resection may be a definitive treatment.

## Acknowledgements

We thank Dr Austin Chen and Dr Savitha Ramachandran for editing the manuscript and for providing critical suggestions.

## Author contributions

All authors have read and agreed to the published version of the manuscript.

**Conceptualization**: Yu-Chi Wang, Yun-Nan Lin.

**Data curation**: Yu-Chi Wang.

**Project administration**: Yu-Chi Wang.

**Supervision**: Yur-Ren Kuo.

**Visualization**: Yu-Chi Wang.

**Writing – original draft**: Yu-Chi Wang, Yun-Nan Lin.

**Writing – review & editing**: Chee-Yin Chai, Hsien-Pin Li, Yi-Ting Chen, Yur-Ren Kuo.
